# Olfactory and Oral Manifestations of COVID-19: Sex-Related Symptoms—A Potential Pathway to Early Diagnosis

**DOI:** 10.1177/0194599820934380

**Published:** 2020-06-16

**Authors:** Ameen Biadsee, Ameer Biadsee, Firas Kassem, Or Dagan, Shchada Masarwa, Zeev Ormianer

**Affiliations:** 1 Department of Otorhinolaryngology–Head and Neck Surgery, Meir Medical Center, Kfar Saba, Israel; 2 Sackler Faculty of Medicine, Tel Aviv University, Tel Aviv, Israel; 3 Department of Oral Rehabilitation, The Maurice and Gabriela Goldschleger School of Dental Medicine, Tel Aviv University, Tel Aviv, Israel; 4 Department of Otorhinolaryngology–Head and Neck Surgery Hillel Yaffe, Medical Center, Hadera, Israel

**Keywords:** coronavirus, COVID-19, anosmia, xerostomia, dysgeusia

## Abstract

**Objective:**

The coronavirus disease 2019 (COVID-19) pandemic poses a threat to global health. Early diagnosis is an essential key to limit the outbreak of the virus.

**Study Design:**

Case series, study conducted between March 25, 2020, and April 15, 2020.

**Setting:**

Ambulatory, nonhospitalized patients who were quarantined in a designated hotel for COVID-19 patients and were recruited by an advertisement at the hotel.

**Subjects and Methods:**

In total, 140 patients participated in a web-based questionnaire assessing initial symptoms of common viral diseases, olfactory and taste functions, xerostomia, and orofacial pain.

**Results:**

A total of 58 men and 70 women participated. Initial symptoms were cough (59.4%), weakness (47.7%), myalgia (46.9%), fever (42.2%), headache (40.6%), impaired sense of smell (38.3%), impaired sense of taste (32.8%), sore throat (26.6%), runny nose (26.6%), and nasal congestion (22.7%). All symptoms were more frequent among women; however, only runny nose was statistically significant (*P* = .018). The most common combination of symptoms was cough and weakness (37.5%). A total of 25.8% reported olfactory and taste dysfunctions in the absence of other symptoms. In a comparison between the sexes, cough and runny nose were the most common combination in women (*P* = .018). A total of 38.3% of patients reported olfactory dysfunction as an initial symptom. Anosmia and facial pain were more common among women (*P* < .001 and *P* = .01, respectively), and 56% of patients reported xerostomia.

**Conclusion:**

A considerable number of patients presented with olfactory and oral disorders. Interestingly, women presented with a different cluster of symptoms than men, which may suggest a new clinical approach to diagnosing COVID-19 disease.

Coronavirus disease 2019 (COVID-19) is an infectious disease caused by a newly discovered coronavirus. The causative pathogen was identified as severe acute respiratory syndrome coronavirus 2 (SARS-CoV-2), which is the seventh type of the coronavirus family to affect humans.^1,2^ On March 11, 2020, the World Health Organization (WHO) declared COVID-19 a global pandemic.

The virus is transmitted from human to human via droplet transmission and direct contact with oral, nasal, and eye mucous membranes.^3^ Studies suggest that COVID-19 may become airborne through aerosols generated during clinical procedures.^4^

COVID-19 has an incubation period of 1 to 14 days, with most ranging from 3 to 7 days.^5^ Other studies suggest an incubation period of 5.2 days.^6^ According to the WHO, an acute respiratory infection, fever, and cough are the most valid diagnostic clinical features.^7^

Social isolation has proven effective in avoiding contamination among the population.^8^ Early detection of symptoms is essential. Some common orofacial manifestations of viral infection may contribute to early diagnosis of COVID-19 infection. Recent reports demonstrated that loss of taste and smell can be the first and only manifestations of infection.^9,10^

This study assessed early manifestations of COVID-19, with an emphasis on olfactory and oral disorders.

## Materials and Methods

The study was conducted in the Department of Oral Rehabilitation, School of Dental Medicine of Tel Aviv University, in collaboration with the Otorhinolaryngology Department, Meir Medical Center (affiliated with Tel Aviv University). The study protocol was approved by the Tel Aviv University Ethics Committee (application number 000120) with digital consent obtained from all participants.

### Participants

According to the Israel Ministry of Health, by April 14, 2020, a total of 9870 COVID-19 patients were diagnosed in Israel (9385 ambulatory patients).^11^ Among them, 140 ambulatory, nonhospitalized patients who were quarantined in a designated hotel for COVID-19 patients were recruited by an advertisement at the hotel (sample size 1.42%). All patients were diagnosed by reverse transcription–polymerase chain reaction assay (RT-PCR) and considered to have mild symptoms, according to the latest WHO joint report.^12^

A web-based survey tool, Google Forms (Google, LLC), was used to create the questionnaire. A standard digitally secured questionnaire link was sent to each patient’s mobile phone. Patients could submit the questionnaire only once. A digital consent to participate in the study was obtained prior to completing the questionnaire.

### Questionnaire

A questionnaire was designed for this study because most available questionnaires did not include the epidemiological and various possible oral, taste, and smell manifestations of COVID-19 infection.

The questionnaire contained 6 sections and a total of 31 questions (see Appendix A in the online version of the article). The first section included demographic data, smoking status, and chronic medication use. The second section contained a multiple-choice question about geographic location of source of infection (Israel, Europe, United States, or other) if known, estimated date of exposure, date of the first symptom, and date of detection by the Israeli health services. Estimated incubation period was calculated as the number of days between the estimated date of exposure and the date of the first symptom, in order to investigate its effect on different variables. In addition, a checklist of common symptoms of viral infection, such as cough, fever, myalgia, weakness, sore throat, nasal congestion and rhinorrhea, dysfunction in olfaction and taste, and headache, was included. The patient could mark more than 1 symptom.

Section 3 contained questions regarding oral hygiene. The question, “Has your dentist advised you that you have gum disease?” was included to determine whether chronic gingivitis and possible oral bleeding were associated with COVID-19 infection.

**Figure 1.**
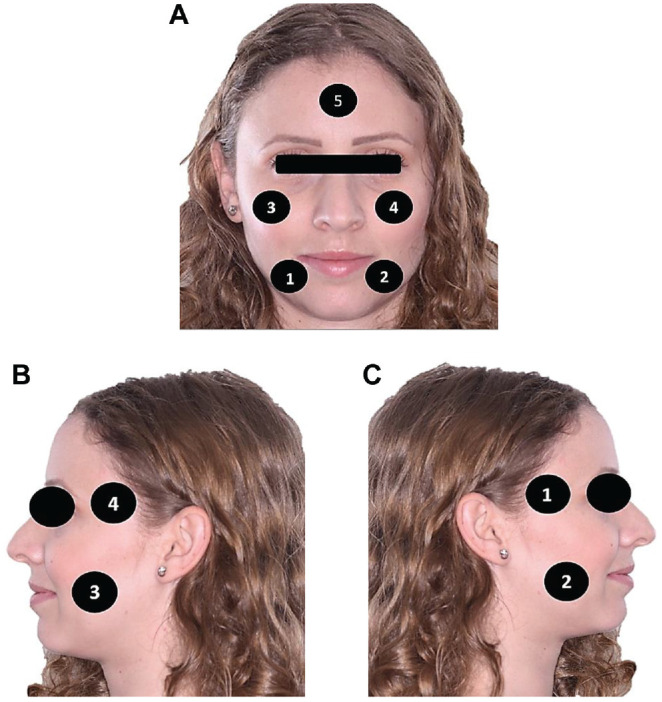
Pain locations (facial and masticatory muscle pain). (**A**) Facial pain. (**B**) Left side masticatory muscle pain. (**C**) Right side masticatory muscle pain.

Section 4 included oral manifestations. It contained simple dichotomous questions about facial pain, masticatory pain, sense of burning in the oral cavity, change in sensation or swelling in the oral cavity, and bleeding. The question, “Do you feel the need to drink more (dry mouth)?” was included to evaluate xerostomia. Pain and burning sensation were estimated using a standard visual analog scale (VAS) pain scale, with 0 representing no pain and 10 severe pain. Patients were asked to mark the locations of facial and masticatory pain (**Figure 1**).

In section 5, taste was assessed using dichotomous questions about spicy, salty, sour, and sweet tastes (taste subgroups). A patient who had experienced a change in any of the tastes was considered to have “taste change.” In section 6, change in smell was evaluated using a dichotomous question. “Is your perception of smells distorted since the onset of the infection, if yes, describe?” was used to describe parosmia, phantosmia, and cacosmia. Anosmia was defined as a score of 0 on the VAS scale. Timing of smell dysfunction was assessed using a multiple-choice question: phase 1 was defined as the first day of illness, phase 2 as third through fifth days, and phase 3 as after the fifth day.

Subjective smell and taste dysfunctions were measured separately using a metric scale, from 0 to 10, with 0 representing anosmia and ageusia and 10 representing a very good sense of smell and taste, respectively. This scale was used to simplify the questionnaire and obtain statistical correlations easily with the VAS scale used with different variables. Information obtained from questionnaires was tallied and summarized.

### Statistical Analysis

Statistical data and figures were analyzed using the R Project for Statistical Computing, version 3.6.2. Reported measures were tested for the association with the demographic variables as follows: sex (man/woman), smoker (yes/no), geographic location of infection (Israel, etc), incubation period (less/more than 5.2 days), and interactions between the latter two. For dichotomous variables such as symptoms (yes/no), their prevalence was fitted in correspondence using logistic regression. Otherwise, linear regression was used. The statistical significance of each covariate was tested using the likelihood ratio test. Correlations between the 11 possible symptoms were analyzed using the Wilk test. Concurrences of any combination of symptoms were further explored with a Venn diagram. The prevalence among men compared to women was tested using Fisher’s odds ratio (OR). All tests and credible intervals are reported at a level of α = 5%.

### Correlation Between Symptoms

A total of 88 unique combinations of symptoms were reported in the sample. Using Wilk’s χ^2^ statistic, we quantified the extent of dependence between the 11 symptoms at once, where

$$\chi^{2}\;=\;651\;\mathrm{with}\;df\;=\;88,\;P\;=\;P\chi^{2}88\;\geq {\;651<\;10}^{-5}$$

indicating extreme dependence.

## Results

Twelve of 140 questionnaires were excluded due to missing information. A total of 58 men and 70 women were included in the study.

Demographic and epidemiological date and initial symptoms are listed in **Table 1**.

**Table 1.** Demographics, Epidemiological Data, and Initial Symptoms.^a^

Characteristic		Value	
Age, mean (range), y		36.25 (18-73)	
Incubation period, mean (range), d		4.6 (1-13)	
Smoking		26 (20)	
Chronic illnesses^b^ (n = 17)			
Asthma		2 (1)	
Hypothyroidism		4 (3)	
Hypertension		8 (6)	
Diabetes mellitus		3 (2)	
Country of infection (n = 128)			
Israel		94 (73)	
European Union		18 (14)	
United States		12 (9)	
Other		2 (2)	
Unknown		2 (2)	
Initial Symptoms^c^	Men	Women	Total
Cough	30 (51)	64 (66)	94 (59)
Weakness	25 (42)	36 (51)	61 (48)
Myalgia	27 (46)	33 (47)	60 (47)
Fever	24 (41)	30 (43)	54 (42)
Headache	21 (36)	31 (44)	52 (40)
Impaired sense of smell	22 (38)	27 (38)	49 (38)
Impaired sense of taste	16 (27)	26 (37)	42 (32)
Sore throat	10 (17)	24 (34)	34 (26)
Runny nose^d^	9 (15)	25 (35)	34 (26)
Nasal congestion	9 (15)	20 (28)	29 (22)
Gastrointestinal symptoms	6 (10)	18 (25)	24 (19)

a Values are presented as number (%) unless otherwise indicated.

b Due to insufficient information, this parameter was not included in statistical analysis.

c Most patients reported more than 1 initial symptom.

d Runny nose was statistically different between the sexes (*P* = .018).

**Figure 2.**
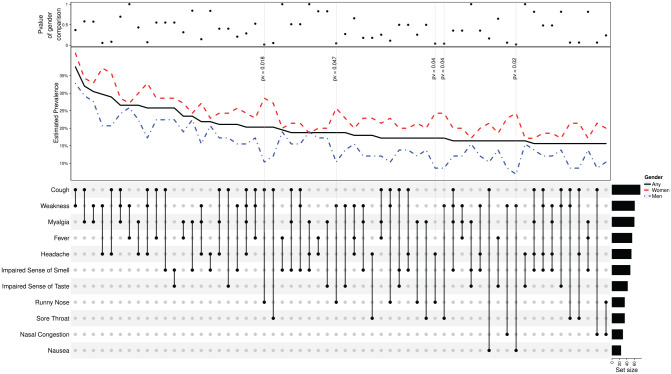
Combinations of initial symptoms. The grid in the lower part maps the combination of symptoms examined (x-axis); the corresponding value on the y-axis is the overall prevalence in the sample (black solid line), prevalence among men (blue dot-dashed line), and prevalence among women (red dashed line). The upper panel reports that Fisher’s odds ratio test for the null odds ratio of men vs women is 1. For example, the far-left column shows the prevalence of patients who experienced both cough and weakness: ≈35% overall, ≈33% of women, and ≈41% of men. Odds ratio with *P*≈ .4. Sixty most frequent combinations are displayed; in 5 combinations, women had significantly increased odds compared to men with *P* < .05 (gray vertical lines).

**Figure 2** illustrates the 60 most prevalent combinations of symptoms among all patients. The most common combinations were cough and weakness (37.5%), cough and myalgia (32%), and myalgia and weakness (30.5%). The top panel shows the corresponding *P* values for comparisons between men and women.

**Figure 3.**
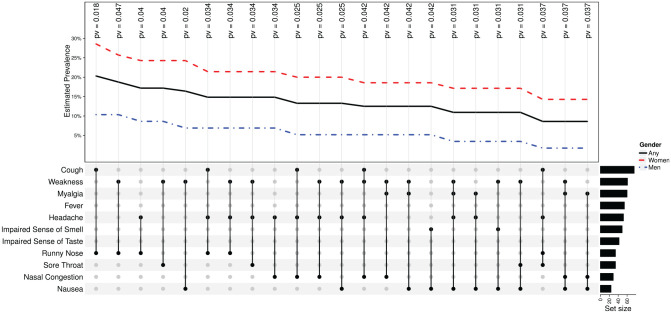
Twenty-three symptom combinations with significant odds ratio (*P* < .05) between men and women. The 128 patients reported 88 unique combinations of symptoms; in 23, there was a significant difference in odds between women and men. The corresponding *P* values are annotated and aligned with each combination.

**Figure 3** illustrates the 23 significant combinations of symptoms between sexes. Cough and runny nose were the most prevalent and significant combination (*P* = .018). It is worth noting that weakness was described in 14 of the 23 most prevalent combinations.

### Olfactory Dysfunction

Eighty-six (67%) patients reported olfactory dysfunction during the disease (35 men, 51 women), and 19.5% reported anosmia, which was more frequent among women (*P* < .001). Mean smell scores after the onset of the disease were significantly lower among women (*P* = .04). Olfactory dysfunction was more common during the third through fifth days of the illness, with no statistical difference between the sexes.

Significant correlations between smell dysfunction, taste dysfunction, and taste subgroups and smell dysfunction were found (*P* < .001). Nasal congestion strongly correlated with smell dysfunction (*P* = .01). However, no correlation with anosmia was demonstrated.

### Taste

Sixty-seven patients (52%) reported changes in taste sensation. Fifty-two patients reported a change in their spicy taste perception, 54 in salty taste, 53 in sour taste, and 61 in sweet taste. In a comparison between men and women, taste change and change in taste subgroups were more common among women, with no significant statistical difference.

Mean scores of taste perception before onset of the disease were higher among women (*P* = .02).

### Dry Mouth

Seventy-two patients reported dry mouth (28 men, 44 women), and a strong association with burning mouth and taste change was found (*P* = .002, *P* = .009, respectively). However, no correlation with any of the taste subgroups, rhinorrhea, or nasal congestion was found.

### Facial Pain

Facial pain was more common among women (*P* = .01), as 18 (26%) reported facial pain, particularly in location 5 (forehead), with mean VAS score of 5 (**Figure 1**).

A significant correlation was found between facial pain and nasal congestion (*P* = .001). This relation was significant in location 5 (*P* = .01). There was no correlation between facial pain and headache (*P* = .08). However, a significant correlation was found between facial pain in location 5 and headache (*P* = .02, *r* = 0.469).

### Masticatory Muscle Pain

Fifteen patients reported muscle pain during mastication (11%), with a mean VAS score of 3.8. Nine patients noted that the pain was in location 3, 7 in locations 1 and 4, and 6 in location 2 (**Figure 1**).

A significant correlation between masticatory and facial pain (*P* < .001, *r* = 0.652) was found.

### Additional Oral Manifestations

Twenty patients reported change in sensation in the tongue, and 9 patients reported plaque-like changes in the tongue. Ten patients reported swelling in the oral cavity: 4 in the palate, 4 in the tongue, and 2 in the gums.

Change in tongue sensation strongly correlated with swollen palate and plaque-like changes in the tongue (*P* < .001).

Six patients reported current oral bleeding, 3 of whom reported past bleeding. No spontaneous bleeding was reported.

### Oral Hygiene

Oral hygiene was not correlated with any of the variables.

## Discussion

### General Symptoms

Coronaviruses are the second most common cause of viral upper respiratory tract infection (URTI) in adults, as well as upper and lower respiratory and systemic symptoms.^13,14^ Previous studies on the symptoms caused by common cold viruses failed to identify the virus based on clinical symptoms, because viral infections per se do not generate symptoms; rather, they are generated by the host’s immune system.^15,16^

**Figure 2** illustrates the 60 most common combination of symptoms among mild COVID-19 patients. Similar to a recently published cohort study, we found that cough, weakness, and myalgia were the most prevalent symptoms.^17^ We found 23 significant combinations of symptoms when comparing men and women (**Figure 3**). These findings are substantially important, because men might have fewer symptoms. An increased level of awareness and caution among health personnel when addressing an asymptomatic patient is mandatory.

### Olfactory Dysfunction

Viral URTI is one of the most commonly identified causes of olfactory dysfunction.^14,18^ Olfactory impairments can be classified into conductive losses stemming from obstruction of the nasal passages and sensorineural causes from damage to the olfactory neuroepithelium, which are most often attributed to postviral olfactory loss.^14,19,20^ Mao et al^21^ reported the presence of neurologic manifestations among COVID-19 patients who were hospitalized in Wuhan, China, in 2019. Smell and taste impairment were the most frequently reported peripheral nerve symptoms. These findings were elucidated based on the known ability of severe acute respiratory syndrome and Middle East respiratory syndrome viruses to enter the central nervous system through a retrograde neuronal route. Since then, several reports have described the same phenomenon.^22,23^ Kaye et al^24^ reported anosmia as the first symptom among more than approximately 25% of COVID-19 patients. Consistent with previous studies, in the current study, 38.3% reported olfactory dysfunction as the first symptom and 66% reported the presence of olfactory dysfunction during the ailment period.

In an effort to find the driving factor for the high prevalence of olfactory dysfunction, we found it significantly correlated with nasal congestion. This implies that obstructed nasal passages might have served as a significant component of the olfactory impairment. Nevertheless, it is worth mentioning that we found no correlation between nasal congestion and anosmia. It is possible that damage to the olfactory neuroepithelium had a meaningful contribution to anosmia.

True loss of taste is extremely rare, and it is usually preceded by the inability to perceive the odor of food due to olfactory dysfunction.^25^ In this analysis, 25.8% reported an impaired sense of smell with impaired sense of taste in the absence of other symptoms. We believe that these results may point out the need to examine and isolate patients with olfactory and taste impairment to reduce the COVID-19 infection rate.

### Xerostomia

Taste is the main stimulant for saliva formation. In our cohort, more than 50% of patients reported dysgeusia and xerostomia, which were significantly correlated, supporting this mechanism. Previous studies showed that xerostomia is secondary to nasal congestion and rhinorrhea due to mouth breathing.^26^ However, there was no significant correlation between dysgeusia and nasal congestion or xerostomia with nasal congestion and rhinorrhea. This condition may be explained by olfactory dysfunction or may suggest neurological involvement that may lead to dysgeusia and xerostomia. Interestingly, we found no significant difference in xerostomia between men and women, which contradicts previous studies in the literature.^27,28^

A common manifestation of xerostomia is a burning sensation, which was demonstrated in this study and confirmed in previous studies.^29^

### Facial Pain

Our findings are in agreement with a number of studies showing that facial pain is more common in women than men.^30,31^ Moreover, a strong correlation between pain in the forehead and headache was found, which can be explained by the patients’ inability to differentiate between headache and facial pain the forehead. Facial pain was associated with nasal congestion, because nasal congestion occurs during URTI as a result of dilatation of veins in the nasal epithelium, adding to the accumulation of secretions in the sinuses.^32^ This leads to pressure changes, eventually stimulating adherent trigeminal nerve endings, causing a pain sensation.^33^

### Limitations

This study was limited by the small sample size. In addition, some data were missing, including information on comorbidities.

## Conclusion

SARS-CoV-2 may manifest with various combinations of symptoms. General symptoms such as cough and weakness (37.5%), cough and myalgia (32%), and myalgia and weakness (30.5%) were the most common combinations of symptoms. This study may provide new clinical information that could increase the ability to diagnose COVID-19 patients sooner. It is especially meaningful to learn about differences between sexes, as cough and runny nose were significantly more common among women than among men. It is important to notice the high proportion of patients who presented only with impaired sense of smell and taste.

## Supplemental Material

supplemental_material_for_Olfactory – Supplemental material for Olfactory and Oral Manifestations of COVID-19: Sex-Related Symptoms—A Potential Pathway to Early Diagnosis Click here for additional data file. Supplemental material, supplemental_material_for_Olfactory for Olfactory and Oral Manifestations of COVID-19: Sex-Related Symptoms—A Potential Pathway to Early Diagnosis by Ameen Biadsee, Ameer Biadsee, Firas Kassem, Or Dagan, Shchada Masarwa and Zeev Ormianer in Otolaryngology–Head and Neck Surgery
