# Supplementary material for: Olfactory and Oral Manifestations of COVID-19: Sex-Related Symptoms—A Potential Pathway to Early Diagnosis
Source: Otolaryngol Head Neck Surg. 2020 Jun 16;163(4):722–8. doi: 10.1177/0194599820934380 (PMC7298562; doi:10.1177/0194599820934380)
Supplement: supplemental_material_for_Olfactory – Supplemental material for Olfactory and Oral Manifestations of COVID-19: Sex-Related Symptoms—A Potential Pathway to Early Diagnosis [file supplemental_material_for_Olfactory.docx]

Appendix A. Study questionnaire

**Section 1. Personal information**

1. Age

1.1. Sex

1.2. Mobile phone

1.3. Do you currently smoke?

a. Yes

b. No

1.4. Do you have any chronic illnesses?

If yes, please note:

1.5. Do you take any medications on daily basis?

If yes, please note:

**Section 2. COVID-19 epidemiology**

2. Country of infection

a. Israel

b. Europe

c. USA

d. Other

3. Date of detection

4. Do you know when you were exposed to COVID-19:

5. When did first symptom appear:

6. What was the first symptom (can be more than one)

a. Cough

b. Fever

c. Muscle pain

d. Runny nose

e. Stuffed nose

f. General weakness

g. Sense of taste affected

h. Sense of smell affected

i. Headaches

j. Nausea/Vomiting/Diarrhea

k. Sore throat

**Section 3. Oral hygiene**

7. how many times a day, do you brush your teeth:

8. How many times a day, do you use mouthwash:

9. How many times a day, do you use dental floss:

10. Do you use any other dental products (Water jet, brushes between teeth, etc.)

11. How often do you visit dental hygienist (in months):

12. Has your dentist advised you, that you have gum disease?

Explain what you were told:

**Section 4. Clinical Oral Manifestation**

4.1 Facial pain

13. Do you have facial pain?

a. Yes

b. No

13.1. If yes, what is the severity on a scale from 0 to 10? **

13.2. If yes, note down numbered areas: (Figure 1.A)

13.3. Have you felt facial pain before COVID-19 onset, if yes how many days before:

4.2 Masticatory muscle pain

14. Do you have facial and masticatory muscle pain?

a. Yes

b. No

14.1. If yes, what is the severity on a scale from 0 to 10? **

14.2 If yes, note down numbered areas (Figure 1.C for the right side and figure 1.B for the left side):

14.3. Have you felt facial and masticatory muscle pain before COVID-19 onset, if yes how many days before?

4.3 Oral cavity

15. Have you experienced any change of sensation in:

a. Tongue

b. Cheeks

c. Gums

d. I do not feel any change of sensation

15.1. Have you felt change of sensation before COVID-19 onset, if yes how many days before?

16. Are you experiencing any swelling in your mouth?

1. Gums 2. Tongue 3. Palate 4. I have no swelling

16.1. Have you felt this swelling before COVID-19 onset, if yes how many days before?

17. Have you noticed any changes in your cheeks or tongue?

a. Yes

b. No

17.1. If Yes, note the changes (color, swelling, texture etc.)

18. Do you have any bleeding in your mouth since COVID-19 onset?

a. Yes

b. No

18.1. If yes, did the exist previously, lately?

a. Yes

b. No

18.2. If yes, when does it occur?

a. When brushing

b. Spontaneously

19. Do you feel the need to drink more (dry mouth)?

a. Yes

b. No

19.1. Have you felt the need to drink more (dry mouth) before COVID-19 onset, if yes how many days before?

20. Are you experiencing a burning sensation in your mouth:

a. Yes

b. No

20.1. If yes, what is the severity on a scale from 0 to 10?

20.2. Have you felt this sensation before COVID-19 onset, if yes how many days before?

**Section 5. Taste**

21. Do you feel a change in your sense of taste- Spicy?

22. Do you feel a change in your sense of taste- salty?

23. Do you feel a change in your sense of taste-sour?

24. Do you feel a change in your sense of taste- sweet?

25. Have you felt these changes before COVID-19 onset, If yes how many days before:

26. Rate your usual sense of taste in general, on a scale from 0 to 10

27. Rate your sense of taste to date, on a scale from 0 to 10

**Section 6. Smell**

28. Have you noticed a change in your sense of smell since COVID-19 onset?

a. Yes

b. No

29.1. Is your perception of smells distorted since COVID-19 onset, if yes describe?

29.2. If your sense of smell has changed, since when?

a. On the first day of illness

b. Between days 3-5

c. After day 5

30. Rate your usual sense of smell in general, on a scale from 0 to 10:

31. Rate your sense of taste to date, on a scale from 0 to 10:
